# Glucagon‐like peptide‐1 receptor antagonism impairs basal exercise capacity and vascular adaptation to aerobic exercise training in rats

**DOI:** 10.14814/phy2.13754

**Published:** 2018-07-08

**Authors:** Rebecca L. Scalzo, Leslie A. Knaub, Sara E. Hull, Amy C. Keller, Kendall Hunter, Lori A. Walker, Jane E. B. Reusch

**Affiliations:** ^1^ Division of Endocrinology University of Colorado School of Medicine Aurora Colorado; ^2^ Department of Medicine Denver VA Medical Center University of Colorado School of Medicine Aurora Colorado; ^3^ Division of Bioengineering University of Colorado School of Medicine Aurora Colorado; ^4^ Division of Cardiology University of Colorado School of Medicine Aurora Colorado

**Keywords:** Aortic strain, mitochondrial respiration, vascular stiffness

## Abstract

Cardiorespiratory fitness (CRF) inversely predicts cardiovascular (CV) mortality and CRF is impaired in people with type 2 diabetes (T2D). Aerobic exercise training (ET) improves CRF and is associated with decreased risk of premature death in healthy and diseased populations. Understanding the mechanisms contributing to ET adaptation may identify targets for reducing CV mortality of relevance to people with T2D. The antihyperglycemic hormone glucagon‐like peptide‐1 (GLP‐1) influences many of the same pathways as exercise and may contribute to CV adaptation to ET. We hypothesized that GLP‐1 is necessary for adaptation to ET. Twelve‐week‐old male Wistar rats were randomized (*n* = 8–12/group) to receive PBS or GLP‐1 receptor antagonist (exendin 9‐39 (Ex(9‐39)) via osmotic pump for 4 weeks ± ET. CRF was greater with ET (*P *< 0.01). Ex(9‐39) treatment blunted CRF in both sedentary and ET rats (*P *< 0.001). Ex(9‐39) attenuated acetylcholine‐mediated vasodilation, while this response was maintained with Ex(9‐39)+ET (*P *= 0.04). Aortic stiffness was greater with Ex(9‐39) (*P *= 0.057) and was made worse when Ex(9‐39) was combined with ET (*P *= 0.004). Ex vivo aortic vasoconstriction with potassium and phenylephrine was lower with Ex(9‐39) (*P *< 0.0001). Carotid strain improved with PBS + ET but did not change in the Ex(9‐39) rats with ET (*P *< 0.0001). Left ventricular mitochondrial respiration was elevated with Ex(9‐39) (*P *< 0.02). GLP‐1 receptor antagonism impairs CRF with and without ET, attenuates the vascular adaptation to ET, and elevates cardiac mitochondrial respiration. These data suggest that GLP‐1 is integral to the adaptive vascular response to ET.

## Introduction

Aerobic exercise capacity is a powerful predictor of cardiovascular (CV) mortality (Wei et al. 1999; Ross et al. 2016) and it is impaired in people with type 2 diabetes (T2D) (Bauer et al. 2007; Regensteiner et al. 2015; Scalzo et al. 2017). Aerobic exercise training improves aerobic exercise capacity, which correlates with decreased risk of premature death. Therefore, understanding the mechanisms that contribute to basal exercise capacity and to the adaptation to aerobic exercise training may identify molecular targets for increasing health span and reducing premature mortality risk in people with T2D. We have reported associations between endothelial dysfunction, increased pulmonary capillary wedge pressure, aortic stiffness, and decreased myocardial perfusion with decreased aerobic exercise capacity in people with T2D (Bauer et al. 2007; Gaspari et al. 2011; Jablonski et al. 2015; Bjornstad et al. 2016). These data suggest a cardiovascular component to the exercise defect in T2D. In addition, recent preclinical data from our group demonstrate a dissociation between skeletal muscle oxygen delivery and distribution in skeletal muscle in metabolic syndrome (Marso and Daniels 2016). Clinical studies in people with T2D demonstrate an increase in skeletal muscle deoxygenation at the onset of exercise in patients with T2D (Bauer et al. 2007; Marso and Daniels 2016) suggesting that blood flow in skeletal muscle is impaired in T2D. Taken together, these findings support a model wherein polyfactorial deficiencies in cardiovascular function and oxygen delivery during exercise contribute to poor aerobic exercise capacity in people with T2D.

Exercise is a classic model of systems biology in action. Cardiac output, determined by the function of the left ventricle, is the first upstream determinant of blood flow delivery to skeletal muscle. The conduit function and reactivity of the vasculature then influence the distribution of the blood flow initiated by cardiac contraction. The interplay of these systems is critical for oxygen delivery to exercising skeletal muscle, and disruption of one component of the system can negatively impact the other. Pharmacological targets that impact the CV system may influence exercise capacity by increasing skeletal muscle blood flow or improving muscle blood flow distribution.

Glucagon‐like peptide‐1 (GLP‐1), an insulin secretagogue, improves glycemic control by improving glucose‐dependent insulin secretion, decreasing glucagon, slowing gastric emptying, and increasing satiety (Mafong and Henry 2009; Monami et al. 2009). In addition to these frequently considered mechanisms of action, GLP‐1 may enhance oxygen delivery to skeletal muscle via augmentation of endothelial and cardiac function in T2D (Ceriello et al. 2006; Mafong and Henry 2009; Koska et al. 2015). Previous investigations using GLP‐1 have demonstrated improvement of left ventricular systolic and diastolic function in humans, rodents, and canines (Luque 2002; Nikolaidis et al. 2004a,b; Nikolaidis 2005; Zhao et al. 2006; Sokos et al. 2007; Mafong and Henry 2009; Scalzo et al. 2017). In the heart and vasculature, GLP‐1 directly stimulates endothelial nitric oxide synthase (eNOS). Studies in rodents, both in vivo and ex vivo*,* suggest a direct effect of GLP‐1 on endothelial function via eNOS activation (Nyström 2008; Keller et al. 2015). In human clinical trials, GLP‐1 recruits muscle microvasculature, lowers blood pressure (Mafong and Henry 2009; Chai et al. 2014), and reduces arterial stiffness (Scalzo et al. 2017). Additionally, large clinical trials have demonstrated the effectiveness of GLP‐1 receptor agonists to decrease premature CV mortality (Marso and Daniels 2016; Steg and Roussel 2016). These observations suggest that GLP‐1 has critical benefits on the CV system of mammals and may augment skeletal muscle and heart blood flow to influence aerobic exercise capacity. In people with T2D, our group reported improvements in measures of cardiac diastolic function and aortic stiffness with exenatide (Scalzo et al. 2017), whereas another group found that liraglutide combined with exercise training resulted in no change in cardiac function (Jørgensen et al. 2017). The interaction of the GLP‐1 receptor function and aerobic exercise training adaptation warrants further investigation as it is a commonly employed and effective treatment for diabetes and obesity.

Our previous work in a rodent model of diabetes demonstrated that increasing circulating GLP‐1 with a dipeptidyl peptidase‐4 (DPP4) inhibitor augments the impact of an aerobic exercise training regimen in a rat model of insulin‐resistant diabetes via augmentation of vascular mitochondrial respiration, content, and ATP production (Keller et al. 2015). To determine if our previous findings were dependent on GLP‐1 receptor signaling, further elucidate the role of GLP‐1 in regard to aerobic exercise capacity, and potentially identify an exercise mimetic, we investigated a GLP‐1 receptor antagonist, exendin 9‐39 (Ex(9‐39)), with and without aerobic exercise training. We hypothesized that GLP‐1 receptor signaling is necessary for cardiac and vascular adaptation to aerobic exercise training.

## Methods

### Animals

Twelve‐week‐old male Wistar rats (Charles River Laboratories International Inc, MA, USA) were randomized to receive phosphate‐buffered saline (PBS; *n* = 8) or Ex(9‐39) (*n* = 12; 250 *μ*g/kg/day, MedImmune, Mountain View, CA, USA) via subcutaneous osmotic pump (2ML4, ALZET, Cupertino, CA, USA) for 4 weeks. Animals in each treatment group were further randomized to remain sedentary or complete 3 weeks of treadmill running. Animals were housed with a 12:12 light cycle and provided water and food ad libitum. The Institutional Animal Care and Use Committee at the Denver VA Medical Center approved all protocols.

### Exercise training

Rats were randomized to remain sedentary or participate in 3 week of nonvoluntary exercise on a Columbus Instruments Exer 3/6 treadmill (Columbus, OH, USA). This training began during the second of the 4 weeks of PBS or Ex(9‐39) delivery. All animals were acclimated to the treadmill over 4 days (sitting stationary on the treadmill for 5 min and increasing to 5 m/min, 0% grade for 10 min). Animals randomized to exercise training completed a 3‐week progressive treadmill running protocol (10–20 m/min, 0–5% grade for 30 min/day).

### Run to fatigue

Aerobic exercise capacity was determined in all animals 24 h after the previous bout of treadmill exercise in the exercise training groups. In the sedentary animals, aerobic exercise capacity was determined 13 days after the treadmill acclimation procedure. All rats ran at the same absolute intensity (18 m/min, 5% grade); fatigue was determined when rats could no longer run or respond to the electric shock plate. The duration of exercise until fatigue was achieved was recorded and distance ran was calculated. Exercise training continued in the rats randomized to exercise for the 3 days following the run to fatigue test to prevent detraining prior to sacrifice.

### Cardiac echocardiography

Echocardiography was performed in rats under anesthesia with 4% isoflurane, immediately prior to sacrifice, using a Vevo 1100 (Visual Sonics Toronto, ON, Canada). The echocardiographer was blinded to treatment groups at the time of measurement as well as during data analysis. Doppler, two‐dimensional (2‐D) and 2‐D‐guided M‐mode images were recorded from parasternal long‐axis and parasternal short‐axis and apical four‐chamber views. Carotid artery diameters during systole and diastole were also measured with the Vevo 1100 at the time of echocardiography. Carotid stiffness was calculated from the change in arterial diameter during the systole–diastole cycle.

### Blood and tissue collection

Animals were anesthetized (for the exercising animals, this occurred 24 h after the last exercise bout) under 4% isoflurane. Blood was collected via cardiac puncture, spun for 10 min at 3600 rpm and 4°C, and plasma was collected and saved at −80°C for glucose (Cayman Chemicals, item #10009582, Ann Arbor, MI, USA) and insulin (ALPCO Diagnostics, Catalogue #80‐INSRT‐E01, Salem, NH, USA) assays.

### Mitochondrial respiration

Mitochondrial respiration was measured using Oxygraph‐2K (O2k,Oroboros Instrument Corporation, Innsbruck, Austria) following methods previously described by our group (Keller et al. 2015, 2016). The heart was removed, and a section of the left ventricle was placed in mitochondrial preservation buffer (BIOPS: 10 mmol/L Ca‐EGTA, 0.1 *μ*mol/L free calcium, 20 mmol/L imidazole, 20 mmol/L taurine, 50 mmol/L K‐MES, 0.5 mmol/L DTT, 6.56 mmol/L MgCl_2_, 5.77 mmol/L ATP, 15 mmol/L phosphocreatine, pH 7.1), and kept on ice. The ventricular tissue was permeabilized via incubation with saponin (50 *μ*g/mL) in BIOPS on ice for 30 min. Then the tissue was washed for 10 min on ice in mitochondrial respiration buffer (MiR06: 0.5 mmol/L EGTA, 3 mmol/L MgCl_2_, 6 mmol/L K‐lactobionate, 20 mmol/L taurine, 10 mmol/L potassium phosphate, 20 mmol/L hEPES, 120 mmol/L sucrose, 1 g/L bovine serum albumin, 280 *μ*g/mL catalase, pH 7.1). The tissue was blotted dry, weighed, and added to prewarmed (37°C) MiR06 in the chamber of the O2k. Oxygen concentration in the MiR06 began at ~400* μ*mol/L and was maintained above 250* μ*mol/L. Respiration was determined by the addition of mitochondrial substrates and inhibitors. Respiration rates were measured with the final concentrations of 5 mmol/L pyruvate + 2 mmol/L malate + 10 mmol/L glutamate (PMG) + 6 mmol/L succinate (PMGS) or 200* μ*mol/L octanoylcarnitine + 1 mmol/L malate (OCM) + 2 mmol/L glutamate + 6 mmol/L succinate (OCMGS), 2 mmol/L adenosine diphosphate (ADP), 10 *μ*g/mL oligomycin, and 0.5* μ*mol/L stepwise titration of carbonyl cyanide 4‐(trifluoromethoxy)phenylhydrazone (FCCP) added until maximal uncoupling (uncoupled state). Mitochondrial membrane integrity was determined by the addition of cytochrome c (10* μ*mol/L).

### Aortic vasoreactivity

Aorta were excised, placed in ice‐cold physiological saline solution, debrided of loose fat and connective tissue and prepared for measurement of isometric force as previously described (Walker et al. 1998; Babu et al. 2004). The thoracic segment of aorta was dissected free from surrounding tissues and cut into rings of 2 mm in length. The preparation was then transferred into organ baths containing Krebs solution (119 mmol/L NaCl, 4.7 mmol/L KCl, 2.5 mmol/L CaCl_2_, 1 mmol/L MgCl_2_, 25 mmol/L NaHCO_3_, 1.2 mmol/L KH_2_PO_4_, 11 mmol/L D‐glucose) bubbled with a mixture of 95% O_2_ and 5% CO_2_. Each aortic ring was mounted between two L‐shaped stainless steel hooks, one of which was connected to a force‐displacement transducer (Grass Instruments Co. WI, USA). Basal tension (1.5 g) was applied to each ring, and all experiments were performed at 37°C. Tissues were constricted with 80 mmol/L KCl and 2 *μ*mol/L phenylephrine. Endothelial‐dependent relaxation was stimulated with 20* μ*mol/L acetylcholine. Data were collected using AcqKnowledge software and were normalized to tissue weight taken at the end of the reactivity protocol.

### Aortic stiffness

Aorta was frozen in PBS at sacrifice. Tissue force‐displacement data were acquired with an MTS Insight 2 mechanical testing system (MTS, Eden Prairie, MN, USA) equipped with a 5‐M load cell and biological environmental chamber. During the tests, the chamber contained calcium and magnesium‐free NaCl‐PBS buffer (0.01 mol/L, ionic strength 0.15, pH 7.4) at 37°C. Sample width, thickness, and gage length were measured using optical methods.

Engineering stress (*σ*) and strain (*ε*) were calculated directly from measured initial values as *σ* = *F*/*WT* and *ε* = (*L* − *L*_0_)/*L* were *F* is the measured force, *W* and *T* are the width and thickness of the sample perpendicular to the load, *L* is the loaded sample length and *L*_0_ is the initial sample (gage) length. Stress–strain curves thus obtained were fit to 9th‐order polynomials to facilitate calculation of incremental elastic moduli (*E*) throughout their strain range, where *E* = *dσ*/*dε*.

### Western blotting

The aortic arch and a section of the left ventricle were flash frozen for determination of protein content of targets of interest. Tissues were homogenized with liquid nitrogen in lysis buffer (MPER (Thermo Scientific (Waltham, MA, USA) with 150 mmol/L NaCL, 1 mmol/L EDTA, 1 mmol/L EGTA, 5 mmol/L Na_4_P_2_O_7_10H_2_O), 1 mmol/L Na_3_VO_4_, 20 mmol/L NaF, 500 mmol/L okadaic acide, 1% protease inhibitor cocktail (Sigma Aldrich, St. Louis MO, USA). Further tissue homogenized occurred utilizing an IKA Ultra‐Turrax T8 Homogenizer (Sigma Aldrich, St. Louis, MO, USA). Lysate protein concentration was determined by the Bradford method and equal protein was resolved on 4–15% SDS‐PAGE gels followed by transfer to PVDF membrane. Membranes were probed with specific antibodies for sirtuin 3 (SIRT3, Cell Signaling # 2627S, 1:1000), peroxisome proliferator‐activated receptor gamma coactivator 1‐alpha (PGC1*α*, Abcam #ab54481, 1:500), total endothelial nitric oxide synthase (eNOS, Cell Signaling #9572S, 1:500), Ser1177 phosphorylated eNOS (Cell Signaling #9571S, 1:500), total 5′ AMP‐activated protein kinase (AMPK, Cell Signaling #2532S, 1:1000), Thr172 phosphorylated AMPK (Cell Signaling #2531S, 1;1000), mitochondrial complexes *I*–*V* subunits (Abcam #ab110412, 1:1000), eukaryotic translation initiation factor 4E‐binding protein 1 (4eBP1, Cell Signaling #9452S, 1;1000), and alpha‐tubulin (Abcam #ab7291, 1:1000). Proteins were detected by fluorescent secondary anitbodies and detected/visulaized by Odyssey CLX (LI‐COR Biotechnologies, Lincoln, NE, USA). Western blot scans and densitometric analysis were performed using Image Studio v4.1. All data are normalized to alpha‐tubulin protein expression.

### Citrate synthase activity

Citrate synthase activity of aortic arch lysates was assayed spectrophotometrically as previously described with slight modifications (Spinazzi et al. 2011) on a BioTek Synergy H1 microplate reader. Enzyme activities were normalized to protein assayed by Bradford's method.

### Statistical analysis

Data were analyzed with two‐way analysis of variance (ANOVA); treatment (PBS and Ex(9‐39)) and exercise (sedentary and trained) were the two factors. Pairwise comparisons were performed using the Tukey test where appropriate. Effect size was calculated for aerobic exercise capacity (the primary variable of interest). The level of statistical significance was set at *P* < 0.05. Data are expressed as mean ± SE.

## Results

### Animal characteristics

Characteristics of the rats on the day of tissue collection are presented in Table 1. Rats randomized to treadmill training had lower body mass compared with the sedentary animals (*P* = 0.02). Plasma insulin concentration measured from blood collected with cardiac puncture was not affected by exercise training (*P* > 0.08) or Ex(9‐39) administration (*P* = 0.6). Plasma glucose concentration was greater in Ex(9‐39)‐treated animals compared with controls (*P* = 0.03). Glucose was not affected by exercise training (*P* = 0.6). All measurements are assessed 24 h after the final bout of exercise in ad libitum fed animals.

**Table 1:** Animal Characteristics

	PBS‐sedentary *n* = 8	PBS‐exercise *n* = 8	Ex(9‐39)‐sedentary *n* = 12	Ex(9‐39)‐exercise *n* = 12
Body mass (g)	443 ± 20	403 ± 14^a^	427 ± 10	404 ± 11^a^
Glucose (mg/dL)	206 ± 13	196 ± 3	232 ± 18^b^	244 ± 20^b^
Insulin (ng/mL)	2.19 ± 0.67	1.98 ± 0.48	2.10 ± 0.29	1.81 ± 0.31

Data are mean ± SEM.

a Main effect of exercise training *P* = 0.05.

b Main effect Ex(9‐39) *P* = 0.03.

### Aerobic exercise capacity

**Figure 1:**
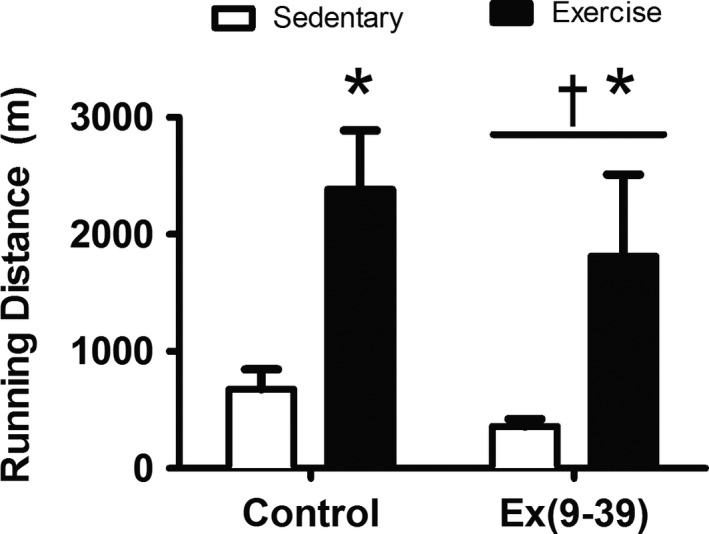
Aerobic exercise capacity. Treadmill run to fatigue distance measured at an absolute intensity. Data are Mean ± SE. *Significant effect of exercise (*P* < 0.01). ^†^Significant effect of Ex(9‐39) (*P* < 0.001). (PBS‐sedentary/exercise trained *n* = 8; Ex(9‐39) sedentary/exercise trained *n* = 12).

Aerobic exercise capacity was determined by distance covered on the treadmill at a fixed intensity. Exercise capacity was significantly greater with treadmill training (Fig. 1; *P* < 0.01). Ex(9‐39) administration attenuated exercise capacity 47% (*P* < 0.001). There was no statistical interaction of exercise training and Ex(9‐39) administration (*P* = 0.14). The effect size for the exercise capacity data was large (*η* ^2^ = 0.8).

### Carotid artery stiffness

**Figure 2:**
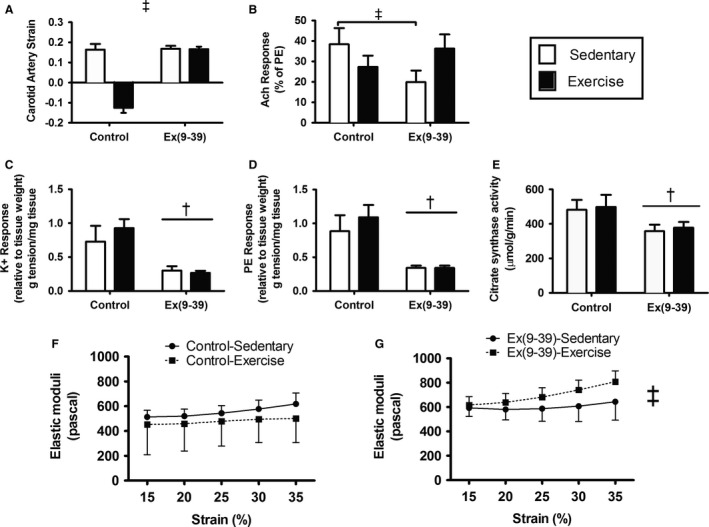
Characterization of the vasculature. (A) Carotid artery strain. (B) Endothelial‐dependent vasodilation in response to acetylcholine (Ach). (C) Vasoconstriction in response to a depolarizing stimulus (potassium, K+). (D) Vasoconstriction in response to a receptor‐mediated stimulus (phenylephrine, PE). (E) Aortic citrate synthase activity. (F) Aortic stiffness measured ex vivo from PBS‐treated rats. (E) Aortic stiffness measured ex vivo from Ex(9‐39)‐ treated rats (Different from PBS control animals (*P* = 0.057)). (F) Aortic citrate synthase activity. Data are Mean ± SE. *Effect of exercise training (*P* < 0.001). ^†^Effect of Ex(9‐39) (*P* < 0.04). ^&^Significant Ex(9‐39) × Exercise interaction (*P* < 0.04). (PBS‐sedentary/exercise trained *n* = 8; Ex(9‐39) sedentary/exercise trained *n* = 12).

Carotid artery stiffness was determined by the relationship of carotid artery diameter during diastole and systole (Fig. 2A). There was a significant interaction of exercise training and Ex(9‐39) (*P* < 0.0001). Exercise training decreased (improved) carotid artery stiffness in the PBS‐treated control animals, whereas Ex(9‐39) abrogated the impact of exercise training on carotid artery stiffness.

### Aortic stiffness

A suggestive increase in aortic stiffness was observed in the Ex(9‐39)‐treated rats (*P* = 0.057); stiffness was not impacted by exercise training (*P* = 0.5). There was a significant interaction of Ex(9‐39) and exercise training on aortic stiffness (Fig. 2F and G; *P* = 0.004). Aortic stiffness was reduced in control animals that were exercise trained, while it was increased in animals exercise trained and treated with Ex(9‐39).

### Aortic vasoreactivity

There was a significant interaction of Ex(9‐39) and exercise training on aortic dilation in response to acetylcholine (Fig. 2B; *P* = 0.04); vasodilation was reduced in Ex(9‐39)‐treated rats that remained sedentary but maintained with the combination of Ex(9‐39) and exercise training. Aortic constriction measured in response to K+, a depolarizing stimulus, and phenylephrine, a receptor‐mediated stimulus, was blunted in aorta from rats treated with Ex(9‐39) compared with control (Fig. 2C and D; *P* = 0.004 and *P* < 0.001). There was no effect of exercise training on the response to K+ (*P* = 0.64) or phenylephrine (*P* = 0.38).

### Aortic citrate synthase activity

Citrate synthase activity was measured in flash‐frozen aortic tissue as a surrogate measure of mitochondrial function. Citrate synthase activity was lower with Ex(9‐39) treatment (Fig. 2E; *P* = 0.01). Exercise training did not affect aortic citrate synthase activity in either group (*P* = 0.71).

### Aortic protein expression

**Figure 3:**
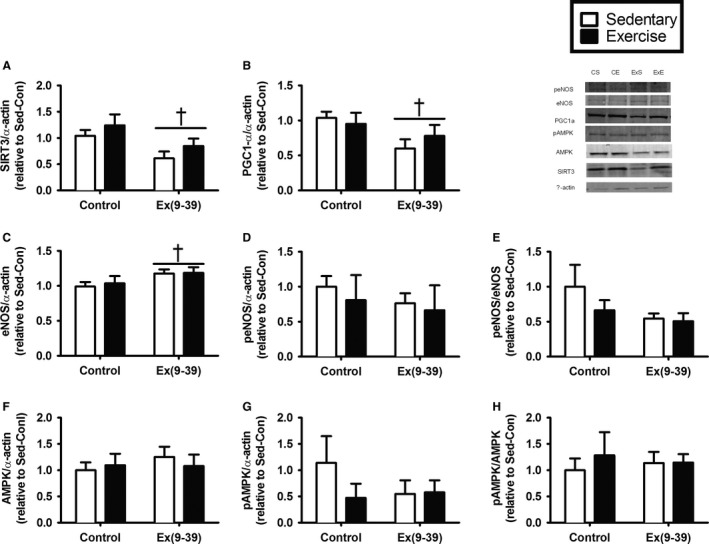
Protein expression in the aorta. (A) Sirt3. (B) PGC1*α*. (C) Total eNOS. (D) Ser1177 phosphorylated eNOS. (E) phospho:total eNOS. (F) Total AMPK. (G) Thr172 phosphorylated AMPK. (H) phospho:total AMPK. Data are Mean ± SE. ^†^Significant effect of Ex(9‐39) (*P* < 0.04). (PBS‐sedentary/exercise trained *n* = 8; Ex(9‐39) sedentary/exercise trained *n* = 12).

Previous work has suggested that upstream signals associated with mitochondrial biogenesis and function are responsive to aerobic exercise training and manipulations of nitric oxide (here through GLP‐1). Therefore, we assessed these signaling proteins in the aorta. Protein expression of SIRT3 and PGC1*α* decreased with Ex(9‐39) treatment (Fig. 3A, *P* = 0.009 and Fig. 3B, *P* = 0.039, respectively). Expression of eNOS increased with Ex(9‐39) administration (Fig. 3C, *P* = 0.04), however, Ser1177 phosphorylation of eNOS and the ratio of Ser1177 phosphorylated to total eNOS did not change with Ex(9‐39) treatment (Fig. 3D, *P* = 0.2 and Fig. 3E, *P* = 0.1, respectively). Exercise training did not alter expression of any of these targets of interest (all *P* > 0.15). Neither Ex(9‐39) administration nor exercise training changed expression of total AMPK (Fig. 3F, *P* > 0.55), Thr 172 phosphorylated AMPK (Fig. 3G, *P* > 0.37), the ratio of Thr 172 phosphorylated to total AMPK (Fig. 3H, *P* > 0.57), or mitochondrial complexes (*P* > 0.29, data not shown).

### Cardiac echocardiography

Parameters characterizing cardiac size and function are presented in Table 2. There were no effects of Ex(9‐39) or exercise on mitral valve E:A or ejection fraction (*P* > 0.08). Administration of Ex(9‐39) increased left ventricular wall diameter and mass (*P* < 0.03). There was no effect of exercise on these measures (*P* > 0.09). Velocity of flow and pressure in the aortic valve were greater with Ex(9‐39) (*P* < 0.04). Neither exercise nor Ex(9‐39) changed other echocardiography measures such as cardiac output or stroke volume (data not shown).

**Table 2:** Cardiac echocardiography

	PBS‐sedentary *n* = 8	PBS‐exercise *n* = 8	Ex(9‐39)‐sedentary *n* = 12	Ex(9‐39)‐exercise *n* = 12
MV E:A	1.54 ± 0.04	1.53 ± 0.04	1.66 ± 0.06	1.69 ± 0.06
Ejection fraction (%)	74 ± 1	76 ± 1	75 ± 3	79 ± 2
LV wall thickness (mm)	2.97 ± 0.18	2.89 ± 0.13	3.53 ± 0.13^a^	3.74 ± 0.16^a^
LV mass (mg)	989 ± 43	908 ± 43	1141 ± 61^a^	1101 ± 57^a^
AV velocity (mm/ms)	873 ± 106	711 ± 16	911 ± 58^a^	983 ± 48^a^
AV pressure (mmHg)	3.14 ± 0.72	2.02 ± 0.09	3.42 ± 0.42^a^	3.93 ± 0.37^a^

Data are mean ± SEM. MV, mitral valve; LV, left ventricle; AV, aortic valve.

a Main effect Ex(9‐39) *P* < 0.04.

### Left ventricular mitochondrial respiration

Mitochondrial respiration from left ventricular samples is presented in Table 3. State 3 respiration with PM and PMGS, uncoupled respiration with PMGS, and the respiratory control ratio with PMGS were greater with Ex(9‐39) compared with control (*P* < 0.02). The ratio of state 2 to state 4 respiration with these substrates was lower with Ex(9‐39) compared with control (*P* < 0.0001). Similar findings were observed in the OCMS experiment for state 3 respiration, uncoupled respiration and the ratio of state 2 to state 4 respiration (*P* < 0.02). There was no effect of exercise training on measures of left ventricular mitochondrial respiration (*P* > 0.1).

**Table 3:** Left ventricular mitochondrial respiration

	PBS‐sedentary *n* = 8	PBS‐exercise *n* = 8	Ex(9‐39)‐sedentary *n* = 12	Ex(9‐39)‐exercise *n* = 12
State 2: PM	53.6 ± 3.3	51.6 ± 2.3	58.4 ± 3.4	49.2 ± 3.4
State 3: PM	171 ± 14	171 ± 17	232 ± 21^a^	302 ± 59^a^
State 3: PMGS	255 ± 23	244 ± 29	346 ± 23^a^	406 ± 53^a^
RCR PMGS	1.77 ± 0.04	1.72 ± 0.06	2.30 ± 0.10^a^	2.41 ± 0.22^a^
Uncoupled: PMGS	293 ± 25	288 ± 29	410 ± 22^a^	444 ± 55^a^
OxPhos ratio: State 2/4	0.49 ± 0.02	0.49 ± 0.02	0.37 ± 0.01^a^	0.39 ± 0.04^a^
State 2: OCM	33.8 ± 3.7	37.3 ± 2.2	32.5 ± 2.6	34.0 ± 2.7
State 3: OCM	109 ± 8	111 ± 7	166 ± 18	133 ± 16
State 3: OCMGS	280 ± 16	235 ± 12	339 ± 29^a^	326 ± 31^a^
RCR OCM	3.35 ± 0.19	3.01 ± 0.08	2.98 ± 0.01	2.95 ± 0.13
Uncoupled: OCMGS	282 ± 15	255 ± 13	351 ± 29^a^	332 ± 27^a^
OxPhos ratio: State 2/4	0.60 ± 0.03	0.56 ± 0.02	0.45 ± 0.01^a^	0.46 ± 0.01^a^

Data are mean ± SEM. P, pyruvate; M, malate; G, glutamate; S, succinate; OC, octanoylcarnitine; RCR, respiratory control ratio.

a Main effect Ex(9‐39) *P* < 0.02.

### Left ventricular protein expression

**Figure 4:**
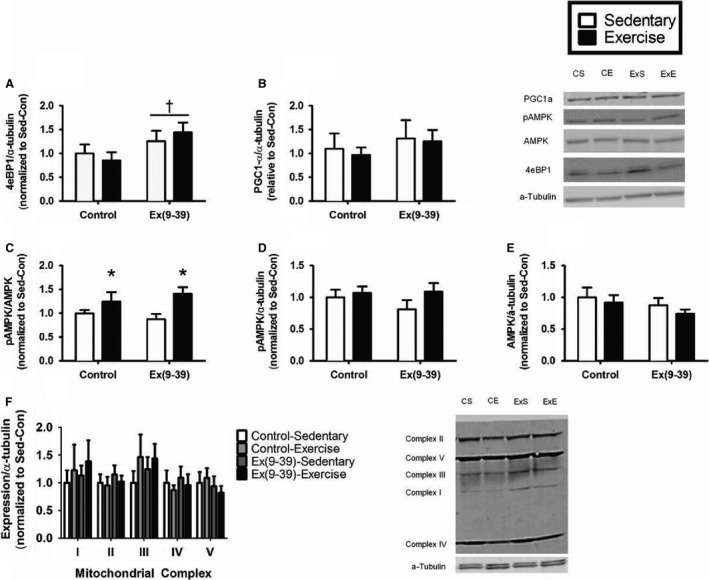
Protein expression in the left ventricle. (A) 4eBP1. (B) PGC1*α*. (C) phospho:total AMPK. (D) Thr172‐phosphorylated AMPK. (E) Total AMPK. (F) Mitochondrial complexes *I*–*V*. Data are Mean ± SE. *Significant effect of exercise (*P* = 0.04). ^†^Significant effect of Ex(9‐39) (*P* < 0.01). (PBS‐sedentary/exercise trained *n* = 8; Ex(9‐39) sedentary/exercise trained *n* = 12).

Due to the changes in cardiac morphology and mitochondrial respiration with Ex(9‐39) administration, we investigated the energetic regulatory status of the left ventricle by measuring proteins associated with cellular energy balance and mitochondrial biogenesis. In the left ventricle, expression of 4eBP1 was greater in animals treated with Ex(9‐39) compared with control (Fig. 4A, *P* < 0.01). There was no effect of exercise on 4eBP1 expression (*P* = 0.93). Activation of AMPK (Thr 172 phosphorylated to total) was greater with exercise training (Fig. 4C, *P* = 0.006). There was no effect of Ex(9‐39) on this metric (*P* = 0.87) and no effect of exercise training or Ex(9‐39) on Thr 172 phosphorylated AMPK (Fig. 4D, *P* > 0.2) or total AMPK (Fig. 4E, *P* > 0.2). There were also no effects of either exercise or Ex(9‐39) on PGC1*α* (Fig. 4B, P > 0.4) or expression of the mitochondrial complexes on the left ventricle (Fig. 4F, *P* > 0.3).

## Discussion

The purpose of these experiments was to determine the necessity of the GLP‐1 receptor on aerobic exercise capacity and simultaneously to define the cardiac and vascular targets of GLP‐1 receptor signaling in the context of exercise training. We found that antagonism of the GLP‐1 receptor with Ex(9‐39) diminishes aerobic exercise capacity, impairs the adaptation of vascular stiffness to exercise training, and alters bioenergetics in the left ventricle of the heart. The changes with Ex(9‐39) in the current investigation were independent of any effect of GLP‐1 receptor antagonism on glycemic control as they were not accompanied by changes in glucose or insulin concentrations at sacrifice. Additionally, the use of an animal model with normal carbohydrate tolerance allowed for the distinction between the impact of glucose and the GLP‐1 receptor antagonism on the endpoints of interest. Together, these findings suggest that GLP‐1 receptor signaling is integral for aerobic exercise capacity and that adaptation to aerobic exercise training in the vasculature is regulated, in part, by GLP‐1 receptor signaling.

Aerobic exercise capacity is our primary variable of interest based upon the strong predictive value of exercise capacity for all‐cause and cardiovascular mortality (Ross et al. 2016), and the well‐established impairment in exercise capacity in people with T2D (Bauer et al. 2007; Regensteiner et al. 2015; Scalzo et al. 2017). We have previously reported an augmentation of exercise capacity (56% increase) with administration of a DPP4 inhibitor combined with exercise training in a rat model diabetes (Keller et al. 2015). These data suggested a potential role of the GLP‐1 receptor to augment adaptation to exercise training. In the current study, antagonism of GLP‐1 receptor signaling, attenuated exercise capacity 47% reflecting the reverse of what we observed previously with the DPP4 inhibitor. Our findings are in agreement with a previous investigation in GLP‐1 receptor null mice where aerobic endurance was lower with the deletion of the GLP‐1 receptor (Ayala et al. 2009). This report adds to the prior finding in the GLP‐1R null mice by examining the adaptive response to exercise training. Together these data suggest that GLP‐1 is important for basal exercise capacity.

The vasculature is one of the many targets of aerobic exercise training. Specifically, aerobic exercise improves measures of vascular function such as reductions in arterial stiffness and augmentation of endothelial‐dependent vasodilation (Lesniewski et al. 2011; Jablonski et al. 2015). In the current study, vehicle‐treated rats exposed to exercise training had lower carotid strain and aortic stiffness compared with the sedentary vehicle‐treated animals. The beneficial effect of exercise on carotid strain was not present in the Ex(9‐39)‐treated rats, and aortic stiffness was greater (worse) in Ex(9‐39)‐treated rats who exercised compared with the sedentary Ex(9‐39)‐treated cohort. The impact of GLP‐1 on vascular function in static conditions (independent of an exercise intervention) was previously appreciated (Nyström 2008; Mafong and Henry 2009; Chai et al. 2014; Keller et al. 2015; Scalzo et al. 2017). Earlier work from our lab suggested a beneficial effect of GLP‐1 on vascular mitochondrial respiration, content, and ATP production (Keller et al. 2015). The data related to carotid and aortic stiffness from the current study confirm the role of GLP‐1 receptor signaling in vascular adaptation to aerobic exercise training.

Independent of the effect of exercise training, we found that aorta from Ex(9‐39)‐treated rats had an impaired vasodilatory response and attenuated vasoconstriction in response to both depolarizing (potassium) and receptor‐mediated (phenylephrine) signals. The decreased vasoconstriction response is suggestive of aortic stiffening, which was confirmed by the measures of elastic moduli in response to increasing strain. Greater aortic stiffness with Ex(9‐39) aligns with data from our previous study in humans with T2D, where we demonstrated that a GLP‐1 receptor agonist (exenatide) decreases aortic stiffness (Scalzo et al. 2017). The functional changes in aortic vasoconstriction and stiffness with Ex(9‐39) were accompanied by a reduction in aortic citrate synthase activity. Together, these data suggest that the vasomotion changes associated with Ex(9‐39) may be due to an impairment in substrate flux through the TCA cycle and potentially attenuated mitochondrial function. This possibility is supported by our previous work with a DPP4‐inhibitor where aortic vasomotion was augmented with treatment in addition to measures of mitochondrial content and function (Keller et al. 2015). The link between GLP‐1 and vascular function has been reported to involve signaling through eNOS, PGC1*α*, and SIRT3 (Gaspari et al. 2011; Monji et al. 2013; Zhao 2013; Wang et al. 2015). In the current study, we observed decreased protein expression of PGC1*α* and SIRT3, regulators of mitochondrial biogenesis and efficiency, and an increase in total eNOS with Ex(9‐39) treatment. The latter finding, increased eNOS protein expression, is possibly a compensation for the decreased expression of PGC1a and SIRT3.

Changes in aortic stiffness, decreased endothelial‐dependent vasomotion, and blunted aortic vasoconstriction with Ex(9‐39) administration were coupled with several measures from the left ventricle often reported concomitantly with hypertension (Palatini et al. 1998). First, left ventricular hypertrophy was present indicated by both increased left ventricular wall thickness and mass with Ex(9‐39) and pressure in the aortic valve was greater suggesting the left ventricle needed to generate greater contractile force to overcome increased afterload secondary to aortic stiffness. Second, State 3 respiration with substrates designed to target both mitochondrial complex I‐ and complex II‐driven respiration was greater in Ex(9‐39)‐treated rats. Additionally, indices of mitochondrial respiration efficiency and capacity were also elevated with Ex(9‐39). We postulate that the left ventricular hypertrophy indices plus the elevation in mitochondrial respiration demonstrate increased demand on the heart as and physiological adaptation for aortic dysfunction mediated by GLP‐1 receptor blockade.

The augmentation of left ventricle mitochondrial respiration in Ex(9‐39)‐treated rats in combination with the morphological changes in the left ventricle with Ex(9‐39) administration lead us to consider the broad molecular signaling associated with cellular energy balance in the left ventricle. To evaluate this possibility, we measured AMPK, a sensor of the ratio of AMP:ATP, and 4eBP1, a marker of mTOR1 activity. Expression of 4eBP1 was greater in the left ventricle of Ex(9‐39)‐treated rats. 4eBP1 is a translation initiation inhibitor, as such its increased expression may suggest that the elevated mitochondrial respiration is fueling contractile work and not synthesis of protein for tissue repair and maintenance (Sonenberg et al. 1996). mTOR signaling is also upstream of mitochondrial activity, and may be a lynchpin of the impact of GLP‐1 on mitochondrial function with exercise in this study. Expression of AMPK was not affected by Ex(9‐39) administration. Given the aortic stiffness created by Ex(9‐39) administration, as well as the impairment in aortic vasoreactivity measured ex vivo, we speculate that given a longer duration of Ex(9‐39) administration, we may have observed the transition from compensatory cardiac hypertrophy with aortic stiffness to pathological remodeling and cardiac dysfunction.

The results of the current study warrant a brief discussion focused on the exercise adaptations present *and* absent in the vehicle‐treated rats. In these animals, we measured a significant increase in exercise capacity with exercise training, the primary variable of interest in this investigation. While this augmentation of exercise capacity was accompanied by improvements in vascular function (specifically carotid strain and aortic stiffness), exercise training did not affect cardiac morphology or mitochondrial respiration in the left ventricle. We had anticipated that we would observe changes in the vehicle‐treated rats representative of cardiac improvement with exercise training based on previous reports (Schreckenberg et al. 2017). Considering the improvement in exercise capacity with training, we now postulate that, in the 12–18‐week‐old Wistar rat, skeletal muscle adaptation coupled with the vascular improvements, account for the increase in exercise capacity observed with exercise training. Additionally, we chose to include only males in this study. Therefore, we are unable to comment on the impact of sex on the findings of the current investigation.

In summary, the GLP‐1 receptor appears to play a crucial role for both endurance exercise capacity and vascular adaptation to aerobic exercise training. The data presented demonstrate the ability of Ex(9‐39) to deleteriously impact both structural and functional properties of the aorta and carotid arteries and to induce left ventricular hypertrophy independent of changes in glucose or insulin. These new preclinical data with Ex(9‐39) treatment identify some plausible mechanisms, whereby GLP‐1 receptor signaling contributes to cardiovascular health. The cardiac changes are similar to those observed in the setting of increased systemic vascular resistance (Palatini et al. 1998) leading us to propose a model wherein GLP‐1 receptor antagonism decreases vascular impedance thereby increasing cardiac workload with resultant physiological hypertrophy. The precise relationship of these cardiac and vascular changes to decreased endurance exercise capacity is not fully resolved by this study.

People with T2D have diminished aerobic exercise capacity, which predicts their increased risk for premature all‐cause and cardiovascular mortality. Of translational relevance, vascular stiffness, impaired endothelial‐mediated vasodilation, and diastolic dysfunction are hallmarks of T2D‐associated CV dysfunction (Zabalgoitia et al. 2001; Diamant et al. 2003; Tabit et al. 2010). Recently, large clinical trials have demonstrated the effectiveness of liraglutide (Marso and Daniels 2016) and semaglutide (Steg and Roussel 2016), GLP‐1 receptor agonists, to decrease premature cardiovascular mortality. We posit that GLP‐1 receptor agonists may also potentiate the beneficial effects of exercise in this population through correction of T2D‐associated vascular dysfunction and call for more work to determine the mechanisms behind this bioactivity. Of particular importance, future research is needed to better understand the long‐term impact of GLP‐1 agonists on cardiac function and the interaction between GLP1 receptor agonists' treatment and exercise (a metabolically effective therapy). Recent physiological studies in T2D demonstrate that each exercise training and GLP‐1 agonists have beneficial effects on preclinical diastolic dysfunction, whereas combination therapy with exercise training and GLP‐1 receptor agonists did not demonstrate this improvement (Jørgensen et al. 2017; Scalzo et al. 2017). These new preclinical data with Ex(9‐39) treatment identify some plausible mechanisms, whereby GLP‐1 receptor signaling contributes to cardiovascular health and suggest future investigation in the clinical setting.

## Conflict of Interest

We have no conflicts of interest to disclose.
